# Emerging Real-World Treatment Patterns and Clinical Outcomes of Multiple Myeloma in Argentina and Brazil: Insights from the TOTEMM Study in the Private Healthcare Sector

**DOI:** 10.3390/curroncol33010016

**Published:** 2025-12-29

**Authors:** Vania Hungria, Angelo Maiolino, Roberto Jose Pessoa de Magalhães, Marcelo Pitombeira de Lacerda, Guillermina Remaggi, Paula Scibona, Cristian Seehaus, Erika Brulc, Nadia Savoy, Dorotea Fantl, Claudia Soares, Gabriela Abreu, Juliana Queiroz, Graziela Bernardino, Straus Tanaka, Mariano Carrizo, Ventura A. Simonovich, Tais Bertoldo Teixeira Fernandes, Bhumika Aggarwal

**Affiliations:** 1 São Germano Clinic, 217—Vila Nova Conceição, São Paulo 04537-081, SP, Brazil; 2 Instituto Oncomed de Educação e Pesquisa, Rua Lopes Trovão, 318, Rio de Janeiro 24220-071, RJ, Brazil; 3 Centro de Hematologia E Oncologia (CHO), R. Alexandre Döhler, Joinville 89201-260, SC, Brazil; 4 Department of Hematology, Fundaleu, Pres. José Evaristo Uriburu 1450, Buenos Aires 1114, Argentina; 5 Hospital Italiano de Buenos Aires, Juan Domingo Peron 4190, Buenos Aires 1199, Argentina; paula.scibona@gmail.com (P.S.); cristian.seehaus@hospitalitaliano.org.ar (C.S.);; 6 GlaxoSmithKline, Estrada dos Bandeirantes 8464, Jacarepaguá, Rio de Janeiro 22783-110, RJ, Brazil; 7 GlaxoSmithKline, Centro Empresarial Libertador, Av. Del Libertador 7208, Buenos Aires 1429, Argentina; mariano.n.carrizo@gsk.com; 8 Orizon, Alameda Tocantins, 822 Barueri, São Paulo 06455-020, SP, Brazil; 9 GlaxoSmithKline, 23 Rochester Park, Singapore 139234, Singapore

**Keywords:** antineoplastic agents, Argentina, Brazil, disease progression, healthcare administrative claims, Latin America, multiple myeloma, real-world, retrospective studies, treatment outcomes

## Abstract

As treatments for multiple myeloma (MM) evolve, there is a need for real-world insights into treatment patterns and outcomes. The **t**reatment practices and clinical **o**utcomes in pa**t**i**e**nts with **MM** (TOTEMM) was a database study (2018–2024) of newly diagnosed transplant-ineligible patients with MM in Argentina (TOTEMM-A) and Brazil (TOTEMM-B) in a private healthcare setting. In TOTEMM-A (n = 72) and TOTEMM-B (n = 892), 37 and 92 different drug regimens were reported, respectively. In each country, treatment duration reduced across lines of therapy (LOT) (TOTEMM-A: range, 6.2–3.4 months; TOTEMM-B: range, 4.4–3.5 months); attrition rates increased across LOT (TOTEMM-A: range, 52.8–86.1%; TOTEMM-B: range, 41.9–88.0%); triplet regimens (mainly bortezomib based) were used most frequently in first-line (1L); >75% relapsed within 12 months, regardless of the drug prescribed; over 90% of relapses occurred between 1L and second-line, and up to half of patients were rechallenged with the same drug; >65% of patients experienced disease progression after 1L; and the 1- to 5-year adjusted cumulative risk of progression or death increased across LOT (TOTEMM-A: range, 47.1–88.5%; TOTEMM-B: range, 40.4–91.7%). The rapid and marked progression underscores the urgent need for novel treatments and regimens.

## Simple Summary

As multiple myeloma (MM) treatments evolve, real-world data is needed to understand how patients are treated and how they respond. The TOTEMM study looked at treatment patterns and outcomes in newly diagnosed MM patients who could not have transplants, using data from private healthcare in Argentina (72 patients) and Brazil (892 patients). Across both countries, many different drug combinations were used, mostly starting with triplet regimens based on bortezomib. Treatment duration shortened with each new line of therapy, while dropout rates increased. Over 75% of patients relapsed within a year, and most relapses happened between the first and second treatment lines. Many were likely to be treated again with the same drug. Around 65% showed disease progression after first-line treatment. The risk of progression or death rose steadily over time. These findings highlight the urgent need for better treatment options for patients with MM.

## 1. Introduction

Multiple myeloma (MM) is a plasma cell disorder that continues to pose a therapeutic challenge due to its relapsing and incurable nature [1]. Median overall survival (OS) is approximately 5 years, with most patients receiving four or more different lines of therapy (LOT) throughout the course of the disease [2]. Treatments aim to prolong the duration of response, which is critical for newly diagnosed patients [3,4]. The ability to achieve and sustain a meaningful response decreases with every LOT, placing emphasis on the first-line (1L) therapy [5].

Recent epidemiological trends indicate a rise in the prevalence, incidence, disease burden, and mortality for patients with MM in Latin America [6,7,8]. Despite global advances in MM treatment that have improved progression-free survival (PFS) and OS [9,10], the MYCLARE study highlighted persistent disparities in MM management in Latin America [11]. Both Argentina and Brazil provide free public healthcare services, but approximately 16% and 25% of their respective populations opt for coverage by private healthcare insurance [12,13]. Those in the private healthcare setting are considered to have better access to diagnosis and treatment than those in the public healthcare setting [11,14,15,16,17].

Stem cell transplant (SCT) remains the standard of care for eligible patients with MM, but many, often older adults or those with significant comorbidities, are ineligible for this treatment [18]. These transplant-ineligible patients require an alternative, long-term treatment strategy that prioritizes quality of life over aggressive treatment [19,20].

Understanding MM treatment patterns in clinical practice through real-world data is essential for informing decisions made by physicians, payers, and regulators. However, such data, particularly concerning treatment practices beyond 1L and longitudinal treatment patterns including LOT for MM, remain relatively scarce in Latin America (including Argentina and Brazil) [21,22,23]. Much of the existing evidence in the region originates from academic institutions or public reference centers, where access to innovative treatments may be limited [11,17,22,24]. To address this evidence gap, we conducted a real-world study in Argentina and Brazil to evaluate current real-world treatment practices and outcomes in newly diagnosed, transplant-ineligible patients with MM in a private healthcare setting across four LOT.

## 2. Methods

### 2.1. Study Design and Study Population

**T**reatment practices and clinical **o**utcomes in pa**t**i**e**nts with **MM** (TOTEMM) was a retrospective, longitudinal database study that evaluated the treatment patterns of patients with MM using electronic medical records (EMRs) from Hospital Italiano de Buenos Aires (HIBA), Argentina (TOTEMM-A: 1 January 2018–31 May 2024), and administrative claims data from Orizon, São Paulo, Brazil (TOTEMM-B: 1 January 2018–28 February 2024). Included were records of patients from the health insurance companies who were privately insured and aged ≥18 years at the index date and had ≥1 MM-related health term (Table S1)/International Classification of Diseases, 10th revision (ICD-10) code (C90/C90.0) and ≥1 core drug specific for MM (Table S2). The index date was defined as a proxy of MM diagnosis and could be the first recorded date of an MM-related health term/ICD-10 code (C90/C90.0) or any MM-related treatment or procedure/exam. Eligible patients must have also had their MM classified as an incident case, defined as being treatment-naive for ≥1 year before the first MM treatment (antineoplastic drug, except for corticosteroids [CSs]). Patients who had previously received a SCT (transplant-eligible) were excluded (i.e., patients who received only antineoplastic drugs as a treatment for MM were included in this analysis and were defined as transplant-ineligible). Patients with MM were followed from the index date until lost to follow-up, end of the study period, or death, whichever came first.

### 2.2. Study Objectives and Variables

The primary objective was to describe treatment practices among transplant-ineligible patients with MM among incident cases, focusing on the LOT (1L; second line [2L], third line [3L], and fourth line [4L]) in a private healthcare community setting in Argentina (TOTEMM-A) and Brazil (TOTEMM-B). Secondary objectives were to describe patient demographics (sex, age at index, and body mass index [BMI] in TOTEMM-A only) and clinical characteristics (duration of follow-up, time to first treatment, time to next treatment [TTNT], duration of treatment, relapse, rechallenge, PFS, and OS) (Table S3).

### 2.3. Data Source and Collection

For TOTEMM-A, EMRs were obtained from HIBA, a leading private hospital in Buenos Aires, Argentina [25]. Among the 16% of the population with private healthcare insurance, more than 175,000 adult members are affiliated with HIBA’s primary insurance provider, Hospital Italiano Medical Care Program [12,25]. The HIBA database reflects patients with private healthcare insurance in Buenos Aires [25]. HIBA offers comprehensive diagnostic and treatment facilities that include more than 40 medical specialties, from primary to tertiary care [25,26]. Patients with MM were identified using relevant health terms (Table S1) related to MM compiled by oncologists from HIBA, who then manually reviewed EMRs to confirm diagnosis.

For TOTEMM-B, administrative health claims data were obtained from Orizon, a database of several private insurers across Brazil. Among the 25% of the population with private healthcare insurance, Orizon covers 59%, encompassing data from 30 million patients, 217,000 health providers, and 14,000 pharmacies [13,27]. The Orizon database reflects patients receiving private care from primary to tertiary levels [27]. Patients with MM were identified using ICD-10 codes (C90, multiple myeloma and malignant plasma cell neoplasms, or C90.0, multiple myeloma).

### 2.4. Data Analysis

Results were interpreted descriptively and considered the country context of treatment availability, healthcare practices, and the population covered by the database. No country-level generalizations were performed. Analyses were performed using the statistical softwares R (Version 4.2.2) and Stata 16 (StataCorp LLC, College Station, TX, USA). All analyses were quality controlled by two independent analysts. For continuous variables, descriptive statistics such as mean, median, standard deviation (SD), interquartile range (IQR), and minimum and maximum values are presented. Categorical variables are presented as absolute counts and percentages. The frequencies of each drug and each combination used were calculated. As the LOT were not explicitly captured through the EMRs and claims data, an algorithm was developed based on the receipt and timing of therapy, along with clinical guidelines, to construct the LOT (Table S4). Therapeutic class combinations related to MM treatment were classified as mono-, doublet, triplet, or quadruplet therapy. For the analysis presented here, monotherapy was defined as the use of a single MM-related drug from one of the drug classes listed in Table S2. Combination therapies were defined as doublet, triplet, and quadruplet therapies when two, three, or four MM-related drugs, respectively, from different classes were prescribed concurrently. Maintenance regimens were reported per the specified LOT rules and were considered part of the treatment line being followed. Kaplan–Meier methodology with 95% confidence intervals (CIs) was used to estimate OS and PFS. Estimated PFS, defined as the time from the beginning of LOT to first progression (new antineoplastic drug or death), and OS, defined as the time from the index date until death (resulting from any cause) The adjusted OS and PFS, including censored time by loss to follow-up, were also evaluated.

## 3. Results

### 3.1. Demographic Characteristics

**Figure 1:**
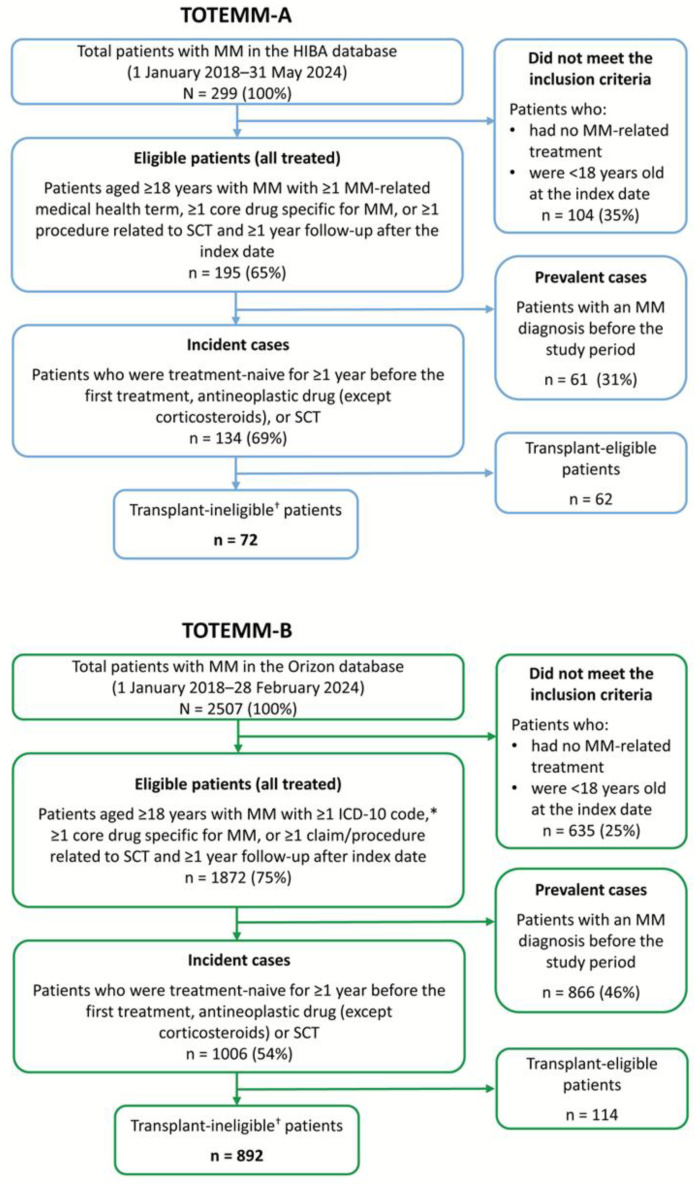
Flowchart of the study populations. * C90, multiple myeloma and malignant plasma cell neoplasms, and C90.0, multiple myeloma. ^†^ Patients who received only antineoplastic drugs as a treatment for MM were included in this analysis. HIBA = Hospital Italiano de Buenos Aires; ICD-10 = International Classification of Diseases, 10th revision; MM = multiple myeloma; SCT = stem cell transplant.

We analyzed data from 72 and 892 transplant-ineligible patients with newly diagnosed MM in Argentina (TOTEMM-A) and Brazil (TOTEMM-B), respectively (Figure 1); their demographic and clinical characteristics are summarized in Table 1. Briefly, in TOTEMM-A, of the 72 transplant-ineligible patients, 41.7% were male, 90.3% were ≥60 years old, and the mean (SD) age at the index date was 76.5 (9.5) years, with a median (IQR) time to follow-up of 19.1 (35.9) months. The proportions of patients declined across LOT: 47.2% in 2L, 31.9% in 3L, and 13.9% in 4L. In TOTEMM-B, of the 892 transplant-ineligible patients, 58.0% were male, 64.6% were ≥60 years old, and the mean (SD) age at the index date was 64.1 (12.3) years, with a median (IQR) time to follow-up of 20.8 (29.7) months. The proportions of patients declined across LOT: 58.1% in 2L, 29.5% in 3L, and 12.0% in 4L.

### 3.2. LOT Duration and Time to Next Treatment

In TOTEMM-A, the median (IQR) LOT durations (months) for 1L, 2L, 3L, and 4L were 6.2 (6.8), 3.8 (9.0), 5.6 (11.7), and 3.4 (9.7), respectively (Table 1). In TOTEMM-B, the median (IQR) LOT durations (months) for 1L, 2L, 3L, and 4L were 4.4 (5.2), 2.8 (8.0), 4.3 (10.6), and 3.5 (10.9), respectively (Table 1).

The median (IQR) times from the index date until first treatment were 0.4 (1.4) and 0.0 (0.6) months in TOTEMM-A and TOTEMM-B, respectively (Table 1). The median (IQR) TTNT decreased in subsequent LOT from 7.8 (7.6) months (1L to 2L) to 6.2 (7.2) months (2L to 3L) and 5.4 (6.0) months (3L to 4L) in TOTEMM-A (Table 1). Similarly, in TOTEMM-B, median (IQR) TTNT decreased from 7.8 (7.4) months (1L to 2L) to 4.6 (7.7) months (2L to 3L) but then increased to 7.2 (10.2) months (3L to 4L) (Table 1).

### 3.3. Treatment Tendencies Across LOT

**Figure 2:**
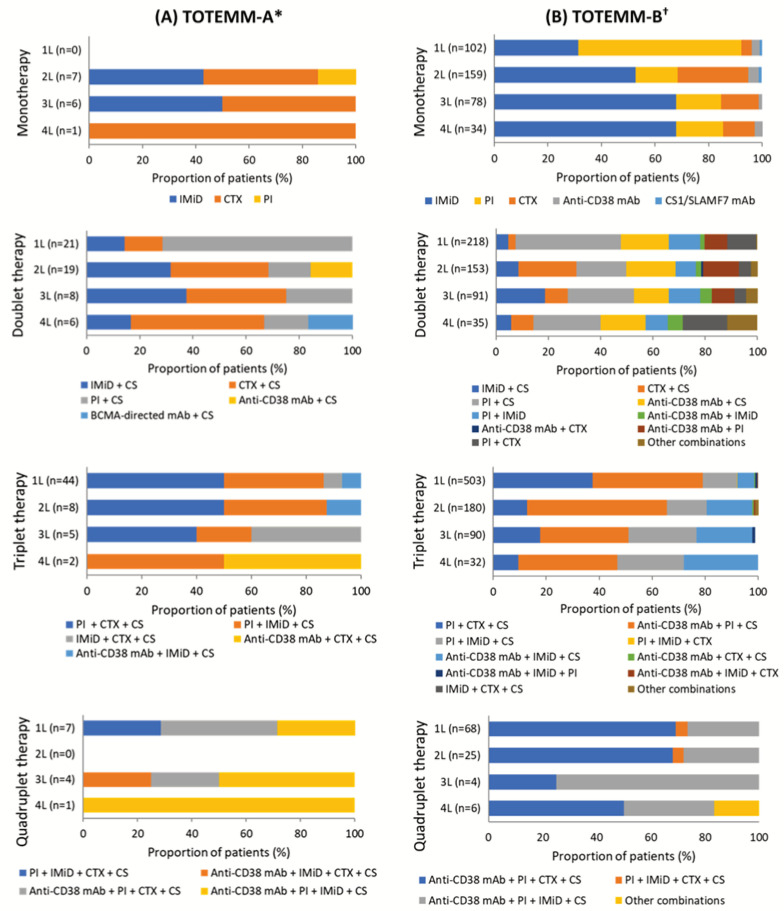
Use of therapeutic classes per LOT in transplant-ineligible patients with MM. The drugs considered to be a CS were dexamethasone, methylprednisolone, prednisone, and prednisolone. * Hospital Italiano de Buenos Aires, Argentina; 1 January 2018–31 May 2024. ^†^ Orizon, Brazil; 1 January 2018–28 February 2024. 1L/2L/3L/4L = first/second/third/fourth line; BCMA = B-cell maturation antigen; CS = corticosteroid; CTX = chemotherapy; IMiD = immunomodulatory drug; mAb = monoclonal antibodies; MM = multiple myeloma; PI = proteasome inhibitor; SLAMF7 = signaling lymphocyte activation molecule family member 7; TOTEMM = **T**reatment practices and clinical **o**utcomes in pa**t**i**e**nts with **MM**.

In TOTEMM-A, among the 72 transplant-ineligible patients, there were 17 different therapeutic class combinations (Table S5) and 37 different drug regimens reported across the study period. The proportions of patients who received mono- (range, 0.0–26.1%), doublet (range, 29.2–60.0%), triplet (range, 20.0–61.1%), and quadruplet (range, 0.0–17.4%) therapy in 1L to 4L varied (Figure S1, Table S5). In 1L, triplet therapy was the most frequently prescribed approach (61.1%; n = 44/72). Among these patients, half (50.0%; n = 22/44) received a combination of a proteasome inhibitor (PI) + chemotherapy (CTX) + CS (Figure 2A, Table S5), with all patients within the combination receiving bortezomib + cyclophosphamide + CS (100.0%; n = 22/22). In 2L, doublet therapy was used most frequently (55.9%; n = 19/34), particularly CTX + CS (36.8%; n = 7/19) (Figure 2A, Table S5). Within this combination, melphalan + CS was most frequent (57.1%; n = 4/7). This pattern continued into 3L, in which doublet therapy remained the most common approach (34.8%; n = 8/23). CTX + CS and immunomodulatory drugs (IMiDs) + CS were equally prevalent (each 37.5%; n = 3/8) (Figure 2A, Table S5), with all patients in these groups receiving cyclophosphamide + CS or lenalidomide + CS (each 100%, n = 3/3). In 4L, doublet therapy remained prevalent (60.0%; n = 6/10), with CTX + CS used in half of these cases (50.0%; n = 3/6) (Figure 2A, Table S5). Among these patients, the most common drug combination was cyclophosphamide + CS (66.7%; n = 2/3). Notably, 83.3% (n = 60/72) of patients received PI-based treatment in 1L (Table S7). Moreover, of all patients who received triplet therapy in 1L to 4L, 83.1% (n = 49/59) received PI-based therapy (Table S5).

In TOTEMM-B, among the 892 transplant-ineligible patients, there were 36 different therapeutic class combinations (Table S6) and 92 different drug regimens reported across the study period in TOTEMM-B. The proportions of patients who received mono- (range, 11.4–31.8%), doublet (range, 24.4–34.6%), triplet (range, 29.9–56.4%), and quadruplet (range, 1.5–7.6%) therapy in 1L to 4L varied (Figure S1, Table S6). In 1L, triplet therapy emerged as the most common approach (56.4%; n = 503/892). Among these patients, 41.6% (n = 209/503) received a combination of anti-CD38 monoclonal antibodies (mAbs) + PI + CS (Figure 2B, Table S6). The most frequently used regimen within this group was daratumumab + bortezomib + CS, accounting for 95.2% of cases (n = 199/209). This pattern continued into 2L, in which triplet therapy remained the most common approach (34.7%; n = 180/518). More than half of these patients (52.8%, n = 95/180) received an anti-CD38 mAb + PI + CS combination (Figure 2B, Table S6), with daratumumab + bortezomib + CS again being the most prevalent (85.3%; n = 81/95). In 3L, the uses of doublet and triplet therapy were nearly equal, at 34.6% (n = 91/263) and 34.2% (n = 90/263), respectively (Figure 2B, Table S6). Among those receiving doublet therapy, the most common regimen was PI + CS (25.3%, n = 23/91) (Figure 2B, Table S6), with carfilzomib + CS used in more than half of these cases (52.2%, n = 12/23). For patients on triplet therapy, one-third (33.3%, n = 30/90) received anti-CD38 mAbs + PI + CS (Figure 2B, Table S6), with daratumumab + bortezomib + CS being the most frequent drug combination (46.7%, n = 14/30). In 4L, doublet therapy was the most frequently prescribed approach (32.7%, n = 35/107), with one-quarter of patients receiving PI + CS (25.7%, n = 9/35) (Figure 2B, Table S6); among them, over half received carfilzomib + CS (55.6%, n = 5/9). Monotherapy in 4L followed closely (31.8%, n = 34/107), with IMiDs being the most frequently used class (67.6%, n = 23/34) (Figure 2B, Table S6); notably, all patients in this group received lenalidomide (100.0%, n = 23/23). Similarly, nearly one-third of patients (29.9%, n = 32/107) received triplet therapy in 4L, with anti-CD38 mAbs + PI + CS being the most common combination (37.5%, n = 12/32) (Figure 2B, Table S6) and daratumumab + carfilzomib + CS the most frequently used regimen within this group (66.7%, n = 8/12). Notably, 85.4% (n = 762/892) of patients received PI-based treatment in 1L (Table S7). Moreover, of all patients who received triplet therapy in 1L to 4L, 87.8% (n = 707/805) received PI-based therapy (Table S6).

### 3.4. Evolving Treatment Tendencies over Time

**Figure 3:**
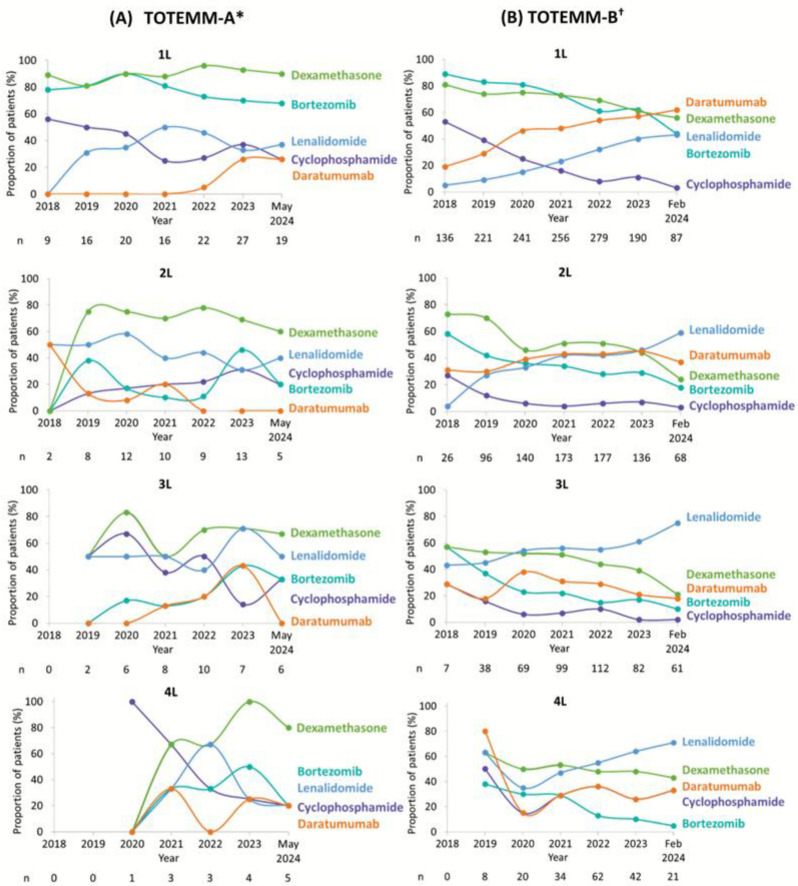
MM-related treatments over time per LOT for transplant-ineligible patients with MM. Due to the study design, patients with newly diagnosed MM (i.e., incident cases) in 2018 would not have had time to receive 3L or 4L treatment; therefore, no patients received 3L or 4L (in TOTEMM-B) in 2018. * Hospital Italiano de Buenos Aires, Argentina; 1 January 2018–31 May 2024. ^†^ Orizon, Brazil; 1 January 2018–28 February 2024. 1L/2L/3L/4L = first/second/third/fourth line; LOT = line of therapy; MM = multiple myeloma; TOTEMM = **T**reatment practices and clinical **o**utcomes in pa**t**i**e**nts with **MM**.

Treatment utilization observations for the most common MM-related treatments in each class across LOT over the study period in TOTEMM-A (1 January 2018–31 May 2024) and TOTEMM-B (1 January 2018–28 February 2024) are shown in Figure 3A,B, respectively.

In TOTEMM-A, 1L usage of dexamethasone and bortezomib was consistently high, whereas cyclophosphamide use declined steadily. Lenalidomide and daratumumab showed increasing uptake over time. In 2L, the use of dexamethasone slightly declined, lenalidomide showed fluctuating use with a downward trend, and cyclophosphamide use slightly increased. Due to the natural course of disease progression, only a small number of newly diagnosed patients with MM in 2018 advanced to 3L or 4L therapy within the same year, resulting in limited availability of data for that period. Moreover, ten or fewer patients per year were included in the analysis for 3L and five or fewer patients per year were included in the analysis for 4L; thus, making any observations is challenging, and no apparent treatment trends are noted.

In TOTEMM-B, in 1L and 2L, there was a clear shift away from bortezomib, dexamethasone, and cyclophosphamide, with all three showing consistent declines in usage. In contrast, daratumumab and lenalidomide usage increased steadily over time. In 3L, a similar downward trend was observed for bortezomib, dexamethasone, cyclophosphamide, and daratumumab, whereas lenalidomide use rose notably, reaching 75.4% by 2024. In 4L, as mentioned above, no observations regarding treatment use in 4L were possible in 2018; thus, data are shown from 2019 onward. A general decline in bortezomib, dexamethasone, cyclophosphamide, and daratumumab was noted, while lenalidomide continued its upward trend.

### 3.5. Relapse and Rechallenge

In TOTEMM-A, among the 33 patients who relapsed during the study period, the median (IQR) time to first relapse (a relapse was considered a progression of LOT; patients should have received ≥2 LOT and ≥1 MM-related core drug) from the start of exposure to bortezomib and/or daratumumab and/or lenalidomide to a subsequent LOT was 7.9 (6.7) months, with 75.8% occurring within 12 months (Table 2). Of these patients, 93.9% (n = 31) experienced relapse at 2L, with a median (IQR) time to relapse of 7.8 (7.3) months. Of the 28 patients exposed to bortezomib, 75.0% (n = 21) relapsed within 12 months of treatment initiation. Among the 11 patients exposed to lenalidomide, 63.6% (n = 7) relapsed within 12 months. Of all three patients exposed to daratumumab, 100.0% (n = 3) relapsed within 12 months (Table 2). Of the number of patients exposed to treatment in 1L who relapsed, rechallenge (use of the same drug at the subsequent LOT) in 2L was reported for 18.5% (n = 5/27) with bortezomib, none with carfilzomib or daratumumab, and 10.0% (n = 1/10) with lenalidomide (Table 3). Attrition rates across LOT were 52.8% from 1L to 2L (n = 38/72), 68.1% from 2L to 3L (n = 11/34), and 86.1% from 3L to 4L (n = 13/23) (Table 1).

In TOTEMM-B, among the 512 patients who relapsed, the median (IQR) time to first relapse from the start of exposure to bortezomib and/or daratumumab and/or lenalidomide to a subsequent LOT was 7.8 (7.2) months, with 75.4% occurring within 12 months (Table 2). The majority of relapses occurred between 1L and 2L. Of the 444 patients exposed to bortezomib, 77.3% (n = 343) relapsed within 12 months of treatment initiation. Among the 85 patients exposed to lenalidomide, 76.5% (n = 65) relapsed within 12 months. Of the 188 patients exposed to daratumumab, 73.9% (n = 139) relapsed within 12 months (Table 2). Of the number of patients exposed to treatment in 1L who relapsed, rechallenge in 2L was reported for 42.7% (n = 189/443) with bortezomib, 35.0% (n = 7/20) with carfilzomib, 44.6% (n = 83/186) with daratumumab, and 53.0% (n = 44/83) with lenalidomide (Table 3). Attrition rates across LOT were 41.9% from 1L to 2L (n = 374/892), 70.5% from 2L to 3L (n = 255/518), and 88.0% from 3L to 4L (n = 156/263) (Table 1).

### 3.6. Estimated Progression-Free Survival

The estimated PFS time was defined as the time from the beginning of LOT to first progression (new antineoplastic drug or death), considering transplant-ineligible patients with MM in Argentina and Brazil. Progression events after 1L, 2L, 3L, and 4L occurred in 69.4% (n = 50/72), 82.4% (n = 28/34), 69.6% (n = 16/23), and 50.0% (n = 5/10) of patients, respectively, in TOTEMM-A, and 66.3% (n = 591/892), 57.7% (n = 299/518), 49.0% (n = 129/263), and 47.7% (n = 51/107), respectively, in TOTEMM-B (Table 4). In TOTEMM-A, the median (P25–P75) estimated survival times (months) of PFS from the start of LOT were 9.7 (4.4–18.9) in 1L, 6.6 (3.7–29.1) in 2L, 9.6 (5.3–14.3) in 3L, and 13.3 (4.6–na) in 4L (Table 4). In TOTEMM-B, the median (P25–P75) estimated survival times (months) of PFS from the start of LOT (excluding the time of censored patients by loss to follow-up) were 10.0 (5.6–23.4) in 1L, 10.5 (3.4–39.6) in 2L, 15.1 (5.6–50.5) in 3L, and 19.2 (4.1–54.2) in 4L (Table 4).

**Figure 4:**
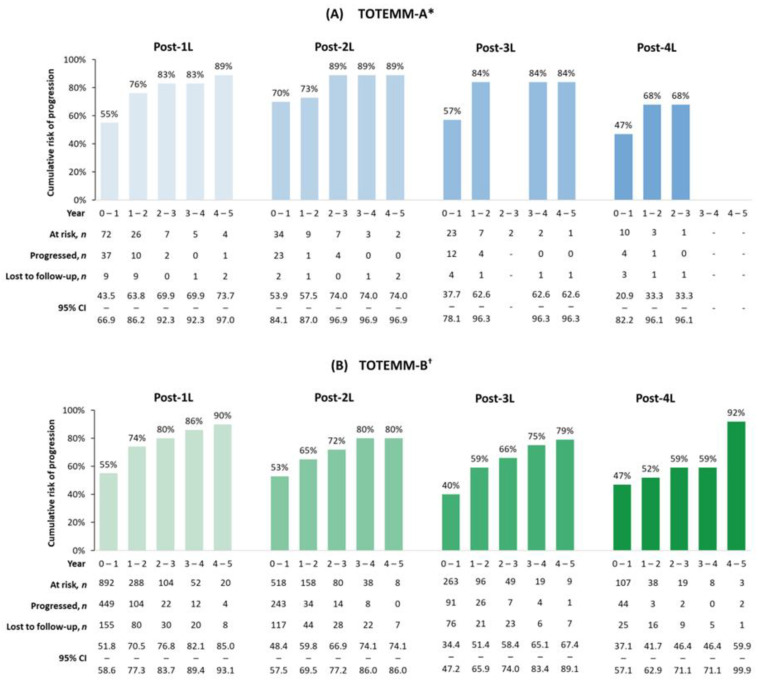
Cumulative risks of progression or death in transplant-ineligible patients with MM. * Hospital Italiano de Buenos Aires, Argentina; 1 January 2018–31 May 2024. ^†^ Orizon, Brazil; 1 January 2018–28 February 2024. 1L/2L/3L/4L = first/second/third/fourth line; CI = confidence interval; MM = multiple myeloma; TOTEMM = **T**reatment practices and clinical **o**utcomes in pa**t**i**e**nts with **MM**.

The 1- to 5-year adjusted cumulative risks of progression or death generally increased across all LOT. In TOTEMM-A (Figure 4A), for 1L, the risk ranged from 54.8% in year 1 to 88.5% by year 5. Similarly, in 2L, risks increased from 69.7% in year 1 to 88.5% by year 5. In the 3L setting, the risk ranged from 57.1% to 83.5%, with an absence of progression events observed at year 3. In 4L, there was an initial lower risk of 47.1% at year 1, with an absence of progression events observed from year 3 onward. In TOTEMM-B (Figure 4B), for 1L, the risk ranged from 55.1% in year 1 to 89.5% by year 5. In 2L treatment, there was a similar upward trend, with risks increasing from 52.9% in year 1 to 80.4% by year 5. In 3L, the risk rose from 40.4% in year 1 to 79.3% by year 5. In 4L, risk ranged from 46.6% in year 1 to 91.7% by year 5. 

### 3.7. Overall Survival

**Figure 5:**
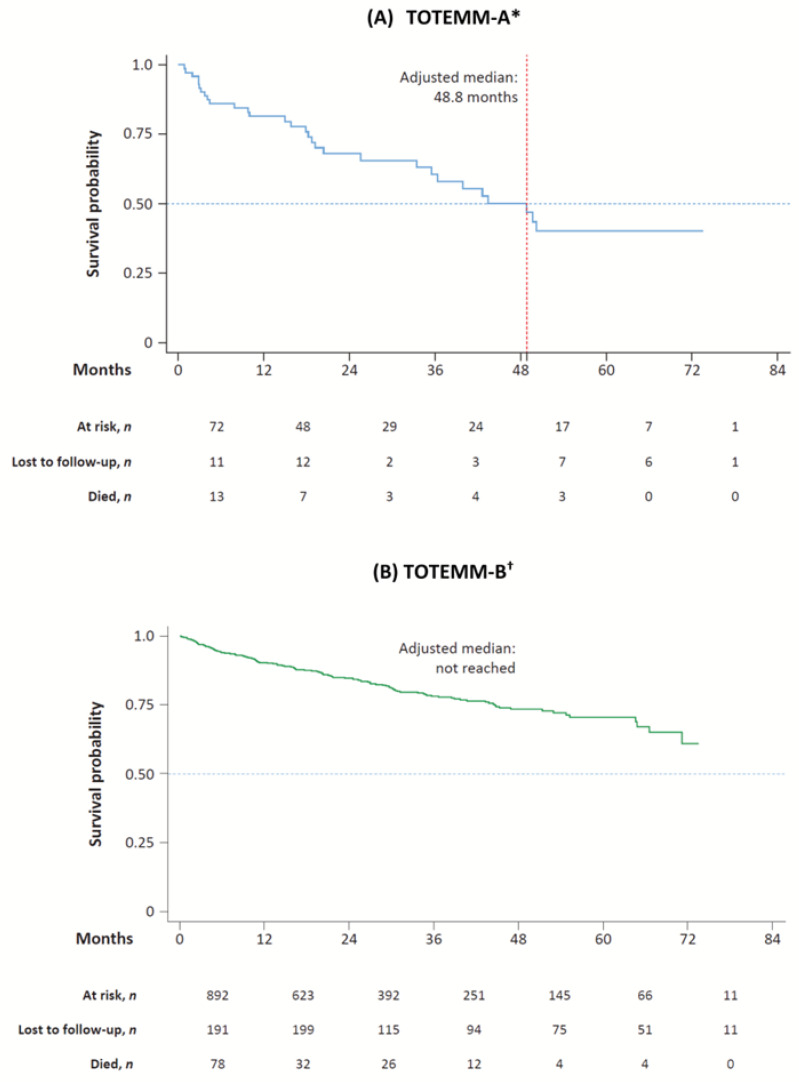
Kaplan–Meier curves for OS of transplant-ineligible patients with MM. * Hospital Italiano de Buenos Aires, Argentina; 1 January 2018–31 May 2024. ^†^ Orizon, Brazil; 1 January 2018–28 February 2024. MM = multiple myeloma; OS = overall survival; TOTEMM = **T**reatment practices and clinical **o**utcomes in pa**t**i**e**nts with **MM**.

Kaplan–Meier curves for adjusted OS estimates among transplant-ineligible patients with MM from Argentina and Brazil are shown in Figure 5.

In TOTEMM-A, the adjusted median OS time was 48.8 months; 30 deaths were registered during the study period (41.7% of 72 incident cases), and the median (IQR) time from index to death was 16.8 (31.9) months (Table S8). The 1- to 5-year cumulative survival rates were 80.5%, 67.0%, 59.9%, 49.2%, and 38.3%, respectively (Table S9).

In TOTEMM-B, the adjusted median OS time was not reached; 156 deaths were registered during the study period (17.5% of 892 incident cases), and the median (IQR) time from index to death was 11.9 (22.3) months (Table S8). The 1- to 5-year cumulative survival rates were 90.2%, 84.7%, 78.1%, 73.5%, and 70.8%, respectively (Table S9).

## 4. Discussion

TOTEMM-A and TOTEMM-B provide valuable insights into current real-world treatment patterns and outcomes (2018–2024) for transplant-ineligible patients with MM within the private healthcare sector in Argentina and Brazil. The studies found that more than 75% of patients experienced disease relapse within 12 months after starting 1L therapy, with most relapses occurring between 1L and 2L, which indicates a consistent pattern of limited durability of response in the frontline setting. There was notable variability in treatment approaches in later LOT, with triplet regimens being prevalent in early LOT. Many patients were rechallenged with the same drug class they received in 1L, particularly in Brazil. The studies highlight the urgent need for more effective and durable therapies, especially at first relapse, to improve patient outcomes and quality of life. Despite advances in treatment, the high attrition rates and progressively shorter survival rates across successive LOT underscore this necessity. Our findings are notable given that patients in the private healthcare system have greater access to novel treatments, compared with patients who rely solely on public healthcare systems [11,13,14,15,28]. This is reinforced by the MYLACRE study, which revealed significant shortcomings in the management of MM in Latin America, with disparities in patient profiles, clinical presentation, treatment approaches, transplant eligibility, and long-term outcomes between the public and private healthcare sectors [11].

We observed variation in treatment patterns across LOT among transplant-ineligible patients in both Argentina and Brazil in the private healthcare setting. According to the TOTEMM-A analysis, triplet regimens were used predominantly in 1L, particularly PI + CTX + CS, with bortezomib-based regimens being most common. Doublet therapy regimens gained prominence in subsequent LOT, particularly CTX + CS and IMiD + CS, reflecting a lack of response to previous LOT and limited access to agents with novel mechanisms of action. The diversity of therapeutic strategies was reflected in the 17 class combinations and 37 unique antineoplastic drug regimens across the study period (January 2018–May 2024), with notable shifts in drug utilization trends. Bortezomib and dexamethasone maintained high usage in 1L, whereas cyclophosphamide use declined. The use of bortezomib-based 1L therapy is consistent with the Haemato-Oncology Latin America (HOLA) observational study, which reported a significant increase in bortezomib-based 1L therapy across Latin America from 2008 to 2015 [6,21]. Lenalidomide and daratumumab showed increasing uptake in later years, particularly in 3L, consistent with global recommendations in MM management [2,29]. Likewise, the diversity of treatments was notable in TOTEMM-B, with 36 therapeutic class variations and 92 distinct regimens reported. In Brazil, as in the TOTEMM-A analysis for Argentina and other real-world studies [9,21], triplet regimens were used most commonly in 1L, notably daratumumab + bortezomib + CS. In later LOT, treatment patterns diversified with a transition toward doublet therapy and monotherapy approaches, often involving lenalidomide or carfilzomib. Over the study period (January 2018–February 2024), there was declining use of bortezomib, dexamethasone, and cyclophosphamide, alongside increased adoption of daratumumab and lenalidomide, reflecting evolving clinical practice and drug availability in Brazil’s private healthcare sector [6,30].

In the TOTEMM-A and TOTEMM-B studies, the high rate (>75%) of early relapse observed with 1L agents, including bortezomib, daratumumab, and lenalidomide, indicates a consistent pattern of limited durability of response in the frontline setting and the need for novel agents and treatment combinations. Among those who experienced a relapse, many patients were likely to be rechallenged using the same antineoplastic drug they received in 1L and 2L. These findings align with those of other real-world studies, [31,32] including a US-based real-world study conducted between 2011 and 2017, which reported that a significant proportion of patients with relapsed/refractory (R/R) MM were re-treated with a regimen containing agents previously used in 1L [33]. Furthermore, the higher rates of rechallenge in 2L observed in TOTEMM-B compared with TOTEMM-A, particularly for lenalidomide and daratumumab, may reflect evolving clinical practice patterns influenced by variations in access to novel treatments or improved tolerability and efficacy perceptions [6,34]. A recent systematic literature review indicated that anti-CD38–based re-treatment provided limited and variable clinical improvements in PFS and OS in patients with R/R MM [35]. Likewise, the LocoMMotion study demonstrated poor clinical outcomes in triple-class–exposed patients (who received at least a PI, IMiD, and anti-CD38 mAb) with R/R MM in a real-world setting, reinforcing the need for therapies with novel mechanisms of action [36].

The high attrition rates (Argentina, 52.8–86.1%; Brazil, 41.9–88.0%) across LOT seen in this study align with those in a US-based study, which reported attrition rates of 43–57%, emphasizing the necessity for enhanced treatment approaches [5,37]. In addition, the declines in treatment duration in Argentina (6.2 months in 1L to 3.4 months in 4L) and Brazil (4.4 months in 1L to 3.5 months in 2L) were steeper than that observed in the US cohort (6.9 months in 1L to 5.7 months in 4L) [5]. Compared with other regions, a potential reason for the shorter treatment duration and high attrition rates observed in Argentina and Brazil may partly be due to limited or delayed access to novel drugs and treatment combinations in the Latin American region [11,30]. Furthermore, the drugs used may differ among healthcare providers, influenced by access constraints and local guidelines, such as those from the Society of Hematology in Argentina (SAH) and the Brazilian Medical Association (AMB) [30,38,39].

In Argentina and Brazil, more than 65% of transplant-ineligible patients with MM experienced disease progression after 1L therapy, compared with 43% in a US-based study [5]. Moreover, high progression rates across later LOT (TOTEMM-A, 50.0–82.4%; TOTEMM-B, 47.7–57.7%) and increasing 1- to 5-year adjusted cumulative risk of progression or death after 1L (TOTEMM-A, 54.8–88.5%; TOTEMM-B, 55.1–89.5%, respectively) suggest limited durability of response and rapid disease progression despite ongoing treatment. The median PFS durations in 1L and 2L in TOTEMM-A (1L, 9.7 months; 2L, 6.6 months) and TOTEMM-B (1L, 10.0 months; 2L, 10.5 months) were generally consistent with those in previous studies from the region. For instance, in the study in Latin America, median PFS times following 1L and 2L were 15.0 and 10.9 months, respectively, in transplant-ineligible patients diagnosed with MM from 2008 to 2015 [21]. In contrast, in the US-based CONNECT Registry real-world study, patients without a transplant with MM from 2009 to 2016 had median PFS durations of 21.5 and 7.3 months after 1L and 2L, respectively [40].

Recent advancements in MM treatment have raised expectations for median OS beyond 5 years in transplant-ineligible patients [2]. Notably, the OS data (median, 48.8 months) from TOTEMM-A fall short of the global 5-year expectations [2], as many studies have reported median OS figures exceeding this benchmark [41,42,43], although these figures still surpass those of Latin America. In contrast, TOTEMM-B OS data (no median OS reached) align with global expectations and exceed OS data from previous studies in the region [11,21,23]. Although speculative, differences in comorbidities may have contributed to the observed outcomes, given that patients in TOTEMM-A (mean, 76.5 years) are notably older than those in TOTEMM-B (mean, 64.1 years). Between 2016 and 2021, the MYLACRE study reported a median OS of 48.7 months for patients in Latin America, representing those from the public and private sectors and those who have or have not undergone SCT [11]. Among transplant-ineligible patients, the median OS decreased to 37.4 months [11]. It is important to note that methodologies for measuring OS can vary; in the MYLACRE study, OS was measured from treatment onset, whereas in the TOTEMM study, OS was measured from diagnosis. Another consideration is the variability in data collection methods; the MYLACRE study involved expert-led data collection [11], whereas the TOTEMM study reflects data from the treated population followed by physicians with various levels of clinical expertise. These variations highlight the different methodologies used to analyze mortality data and reinforce the importance of contextualizing survival data from the literature.

Since the databases used were not designed primarily for research purposes, the analyses may have been limited by the variables available within them. Notably absent from these datasets were clinically relevant variables such as cancer stage classification, cytogenetic or molecular testing results, and laboratory values, including renal function. Moreover, the potential reasons for a patient’s transplant ineligibility (e.g., frailty score, social support assessment, patient refusal, financial hardship and/or associated comorbidities), loss of follow-up (e.g., switching to a different insurer not included in the database, a change to public healthcare, transferring care to a different hospital, or true cessation of healthcare utilization), and cause of death were not captured in these databases. Clinical care information from outside oncology clinics may be missing or underreported, potentially resulting in misclassification of treatments and outcomes. Of note, although the number of deaths was reported in this study, owing to the nature of the data sources, not all death events were necessarily captured and may be underrepresented. For example, a patient who may have died at home, whose death was not captured in the system, was censored in the dataset instead. As thalidomide is offered in the public setting, its use was not captured in the TOTEMM-B private database setting. Other limitations of the study are typical of retrospective analyses, including those related to the quality of the database, availability of relevant clinical information, and accurate coding of diagnoses, procedures, and medications. Any potential coding inaccuracies may have led to misidentification of cases. To address this limitation, for TOTEMM-A data, oncologists at HIBA manually reviewed the extracted data to confirm diagnoses and verify treatments. In Brazil, healthcare providers are not required to include ICD-10 codes for procedures. As a result, a manual evaluation was conducted on procedures, exams, and treatments related to MM that lacked an ICD-10 code. Moreover, all analyses were quality controlled by two independent analysts. Direct comparisons between the Argentinian and Brazilian data were not performed, as the study was not statistically powered for such analyses, and the databases differed in the context of the source; however, all algorithms used to identify the number of deaths, LOT transitions, and censoring rules were standardized for use in both datasets. Although generalizability is limited to the overall population within the private healthcare setting, the study provides valuable insights relevant to this region.

## 5. Conclusions

Our findings demonstrate high progression, relapse, and attrition rates among transplant-ineligible patients with MM in Argentina and Brazil, with most progression events occurring within 6 months across LOT. Across all LOT, the adjusted cumulative risks of progression and death consistently rose over the 1- to 5-year period. Despite treatment advances, relapse within 1 year of starting 1L therapy was common, particularly between 1L and 2L. Use of triplet regimens, especially anti-CD38–based combinations, was prevalent in early LOT. Treatment variability and frequent rechallenge with the same drug class were notable, and fewer patients reached later LOT over time. These findings highlight the urgency for novel, more effective, durable therapies at first relapse to improve patient outcomes and quality of life. This knowledge is particularly valuable as we navigate the increasingly complex and rapidly evolving treatment landscape for MM, providing essential evidence-based insights for advancing patient care as clinicians gain more experience with novel antineoplastic drugs.

## Figures and Tables

**Table 1:** Demographic and Clinical Characteristics of Transplant-Ineligible Patients With MM.

	TOTEMM-A * N = 72	TOTEMM-B ^†^ N = 892
Sex, n (%)	72	512 **^‡^**
Male	30 (41.7)	297 (58.0)
Female	42 (58.3)	215 (42.0)
Age group at index date, n (%)	72	512 **^‡^**
18–49 years	1 (1.4)	78 (15.2)
50–59 years	6 (8.3)	103 (20.1)
≥60 years	65 (90.3)	331 (64.6)
Age at index date, years, mean (SD)	76.5 (9.5)	64.1 (12.3)
Index year, n (%)	72	892
2018	12 (16.7)	161 (18.0)
2019	15 (20.8)	162 (18.2)
2020	11 (15.3)	141 (15.8)
2021	6 (8.3)	165 (18.5)
2022	13 (18.1)	156 (17.5)
2023	13 (18.1)	99 (11.1)
2024	2 (2.8)	8 (0.9)
Time to follow-up, months, median (IQR)	19.1 (35.9)	20.8 (29.7)
Number of patients per LOT, n (%)		
1L	72 (100.0)	892 (100.0)
2L	34 (47.2)	518 (58.1)
3L	23 (31.9)	263 (29.5)
4L	10 (13.9)	107 (12.0)
LOT duration, months, median (IQR)		
1L	6.2 (6.8)	4.4 (5.2)
2L	3.8 (9.0)	2.8 (8.0)
3L	5.6 (11.7)	4.3 (10.6)
4L	3.4 (9.7)	3.5 (10.9)
Time from index date to first treatment, months, median (IQR) ^#^	0.4 (1.4)	0.0 (0.6)
TTNT, months, median (IQR)		
1L to 2L	7.8 (7.6)	7.8 (7.4)
2L to 3L	6.2 (7.2)	4.6 (7.7)
3L to 4L	5.4 (6.0)	7.2 (10.2)
BMI, n ^§,¶^	70	
Mean (SD), kg/m^2^	26.8 (4.5)	-
Underweight, <18.5 kg/m^2^, n (%)	2 (2.9)	-
Normal, ≥18.5 to <25 kg/m^2^, n (%)	21 (30.0)	-
Overweight, ≥25 to <30 kg/m^2^, n (%)	34 (48.6)	-
Obese, ≥30 kg/m^2^, n (%)	13 (18.6)	-

* Hospital Italiano de Buenos Aires, Argentina; 1 January 2018–31 May 2024. ^†^ Orizon, Brazil; 1 January 2018–28 February 2024. ^‡^ Missing data: 380 patients for Brazil. ^#^ Patients with zero time indicate that their follow-up started on the same date as their first antineoplastic drug treatment. ^§^ Data not available for Brazil. ^¶^ Missing data for 2 patients. 1L/2L/3L/4L = first/second/third/fourth line; BMI = body mass index; IQR = interquartile range; LOT = line of therapy; MM = multiple myeloma; SD = standard deviation; TTNT = time to next treatment.

**Table 2:** Relapse exposure across LOT in transplant-ineligible patients with MM.

	TOTEMM-A *			TOTEMM-B ^†^		
		LOT in Which Relapse ^‡^ Occurred			LOT in Which Relapse ^‡^ Occurred	
	All Patients Who Relapsed	2L	3L	All Patients Who Relapsed	2L	3L
Time to first relapse from the start of exposure, ^§^ n, months	33	31	2	512	508	4
Median (IQR)	7.9 (6.7)	7.8 (7.3)	6.7 (4.2)	7.8 (7.2)	7.8 (7.2)	8.3 (4.6)
Categorical time, n (%)						
0 to ≤2	2 (6.1)	2 (6.5)	-	41 (8.0)	40 (7.9)	1 (25.0)
>2 to ≤6	10 (30.3)	9 (29.0)	1 (50.0)	134 (26.2)	134 (26.4)	-
>6 to ≤12	13 (30.4)	12 (38.7)	1 (50.0)	211 (41.2)	208 (40.9)	3 (75.0)
>12	8 (24.2)	8 (25.8)	-	126 (24.6)	126 (24.8)	-
Exposure to bortezomib, n (%), months ^¶^	28	27	1	444	443	1
0 to ≤2	2 (7.1)	2 (7.4)	-	38 (8.6)	37 (8.4)	1 (100.0)
>2 to ≤6	8 (28.6)	7 (25.0)	1 (100.0)	117 (26.4)	117 (26.4)	-
>6 to ≤12	11 (39.3)	11 (39.3)	-	188 (42.3)	188 (42.4)	-
>12	7 (25.0)	7 (25.0)	-	101 (22.7)	101 (22.8)	-
Exposure to daratumumab, n (%), months ^¶^	3	3	-	188	186	2
0 to ≤2	-	-	-	9 (4.8)	8 (4.3)	1 (50.0)
>2 to ≤6	3 (100.0)	3 (100.0)	-	42 (22.3)	42 (22.6)	-
>6 to ≤12	-	-	-	88 (46.8)	87 (46.8)	1 (50.0)
>12	-	-	-	49 (26.1)	49 (26.3)	-
Exposure to lenalidomide, n (%), months ^¶^	11	10	1	85	83	2
0 to ≤2	1 (9.1)	1 (10.0)	-	3 (3.5)	3 (3.6)	-
>2 to ≤6	2 (18.2)	2 (20.0)	-	23 (27.1)	23 (27.7)	-
>6 to ≤12	4 (36.4)	3 (30.0)	1 (100.0)	39 (45.9)	37 (44.6)	2 (100)
>12	4 (36.4)	4 (40.0)	-	20 (23.5)	20 (24.1)	-

In TOTEMM-A and TOTEMM-B, no patients had relapsed in 4L. * Hospital Italiano de Buenos Aires, Argentina; 1 January 2018–31 May 2024. ^†^ Orizon, Brazil; 1 January 2018–28 February 2024. ^‡^ A relapse was considered a progression of LOT; patients should have received ≥2 LOT and ≥1 core drug (Table S2). ^§^ Time from the start of one LOT to the next. ^¶^ The analysis was restricted to patients exposed to bortezomib, daratumumab, or lenalidomide. 1L/2L/3L/4L = first/second/third/fourth line; IQR = interquartile range; LOT = line(s) of therapy; MM = multiple myeloma; TOTEMM = **T**reatment practices and clinical **o**utcomes in pa**t**i**e**nts with **MM**.

**Table 3:** Frequency of rechallenge * after relapse ^†^ across LOT in transplant-ineligible patients with MM.

	1L		2L			3L			4L
	No. of Patients Exposed in 1L	No. of Patients Exposed to 1L Who Relapsed	Rechallenge in 2L	No. of Patients Exposed to 2L	No. of Patients Exposed to 2L Who Relapsed	Rechallenge in 3L	No. of Patients Exposed to 3L	No. of Patients Exposed to 3L Who Relapsed	Rechallenge in 4L
**TOTEMM-A, ^‡^ n (%)**									
Bortezomib ^¶^	60	27 (45.0)	5 (18.5)	9	4 (44.4)	2 (50.0)	7	2 (28.6)	-
Carfilzomib ^¶^	-	-	-	2	2 (100.0)	1 (50.0)	1	-	-
Daratumumab ^¶^	8	3 (37.5)	-	4	2 (50.0)	1 (50.0)	4	2 (50.0)	-
Lenalidomide ^¶^	24	10 (41.7)	1 (10.0)	13	8 (61.5)	3 (37.5)	10	4 (40.0)	-
**TOTEMM-B, ^§^ n (%)**									
Bortezomib ^¶^	718	443 (61.7)	189 (42.7)	199	107 (53.8)	13 (12.1)	66	30 (45.5)	10 (33.3)
Carfilzomib ^¶^	38	20 (52.6)	7 (35.0)	59	32 (54.2)	19 (59.4)	61	26 (42.6)	7 (26.9)
Daratumumab ^¶^	378	186 (49.2)	83 (44.6)	211	112 (53.1)	36 (32.1)	77	34 (44.2)	11 (32.4)
Lenalidomide ^¶^	196	83 (42.3)	44 (53.0)	182	52 (28.6)	28 (53.8)	129	45 (34.9)	18 (40.0)

Only data for patient numbers >5 for each treatment are shown. * Rechallenge was defined as use of the same drug in the subsequent LOT. ^†^ A relapse was considered a progression of LOT; patients should have received ≥2 LOT and ≥1 core drug (Table S2). ^‡^ Hospital Italiano de Buenos Aires, Argentina; 1 January 2018–31 May 2024. ^§^ Orizon, Brazil; 1 January 2018–28 February 2024. ^¶^ An antineoplastic drug could have been used alone or in combination. LOT = line(s) of therapy; MM = multiple myeloma; TOTEMM = **T**reatment practices and clinical **o**utcomes in pa**t**i**e**nts with **MM**.

**Table 4:** Survival analysis for estimated PFS per LOT of transplant-ineligible patients with MM.

	1L		2L		3L		4L	
	TOTEMM-A N = 72	TOTEMM-B N = 892	TOTEMM-A N = 34	TOTEMM-B N = 518	TOTEMM-A N = 23	TOTEMM-B N = 263	TOTEMM-A N = 10	TOTEMM-B N = 107
Estimated PFS from the start of LOT, n (%)								
Progression	50 (69.4)	591 (66.3)	28 (82.4)	299 (57.7)	16 (69.6)	129 (49.0)	5 (50.0)	51 (47.7)
Up to drug treatment	34 (68.0)	518 (87.6)	23 (82.1)	263 (88.0)	10 (62.5)	107 (82.9)	3 (60.0)	35 (68.6)
Up to death	16 (32.0)	73 (12.4)	5 (17.9)	36 (12.0)	6 (37.5)	22 (17.1)	2 (40.0)	16 (31.4)
Loss to follow-up	22 (30.6)	301 (33.7)	6 (17.6)	219 (42.3)	7 (30.4)	134 (51.0)	5 (50.0)	56 (52.3)
Survival time of PFS from the start of LOT, months, median (P25–P75)	9.7 (4.4–18.9)	10.0 (5.6–23.4) *^,†^	6.6 (3.7–29.1)	10.5 (3.4–39.6) *^,‡^	9.6 (5.3–14.3)	15.1 (5.6–50.5) *^,§^	13.3 (4.6–na) ^¶^	19.2 (4.1–54.2) *^,II^

* Excluding the time of censored patients by loss to follow-up. ^†^ One patient progressed to death on the same date who started the 1L, and one patient was lost to follow-up on the same date, as the start of 1L was not included. ^‡^ One patient was lost to follow-up on the same date as the start of 2L and was not included. ^§^ Two patients were lost to follow-up on the same date as the start of 3L and were not included. ^¶^ No value for P75. ^II^ One patient was lost to follow-up on the same date as the start of 4L and was not included. 1L/2L/3L/4L = first/second/third/fourth line; LOT = line(s) of therapy; MM = multiple myeloma; P25/75 = percentile 25/75; PFS = progression-free survival; TOTEMM = **T**reatment practices and clinical **o**utcomes in pa**t**i**e**nts with **MM**.

## Data Availability

Anonymized subject-level data used for this publication were obtained from the Hospital Italiano de Buenos Aires, Buenos Aires, Argentina, and Orizon, São Paulo, Brazil. GSK was accountable for the statistical analysis of the study. The original contributions presented in this study are included in the article/Supplementary Material. Further inquiries can be directed to the corresponding author.

## Supplementary Materials

The following supporting information can be downloaded at: https://www.mdpi.com/article/10.3390/curroncol33010016/s1, Table S1: Health terms related to MM; Table S2: Treatment agents indicated for MM treatment; Table S3: Variables related to treatment outcomes; Table S4: Algorithm for identifying LOT based on treatment dates and clinical guidelines; Table S5: 1L–4L treatments in transplant-ineligible patients with MM among incident cases in TOTEMM-A; Table S6: 1L–4L treatments in transplant-ineligible patients with MM among incident cases in TOTEMM-B; Table S7: Core 1L–4L MM drug use in transplant-ineligible patients with MM among incident cases in TOTEMM-A and TOTEMM-B; Table S8: Mortality and OS of transplant-ineligible patients with MM among incident cases; Table S9: OS of transplant-ineligible patients with MM for all incident cases per year of follow-up; Figure S1: Treatment combinations across LOT for transplant-ineligible patients with MM.

## Abbreviations

The following abbreviations are used in this manuscript:

1L/2L/3L/4L	first/second/third/fourth line
BMI	body mass index
CI	confidence interval
CS	corticosteroid
CTX	chemotherapy
EMR	electronic medical record
HIBA	Hospital Italiano de Buenos Aires
ICD-10	International Classification of Diseases, 10th revision
IMiD	immunomodulatory drug
IQR	interquartile range
LOT	line(s) of therapy
mAb	monoclonal antibody
MM	multiple myeloma
OS	overall survival
PFS	progression-free survival
PI	proteasome inhibitor
R/R	relapsed/refractory
SCT	stem cell transplant
SD	standard deviation
TOTEMM	**T**reatment practices and clinical **o**utcomes in pa**t**i**e**nts with **MM**
TTNT	time(s) to next treatment
