# Supplementary material for: Emerging Real-World Treatment Patterns and Clinical Outcomes of Multiple Myeloma in Argentina and Brazil: Insights from the TOTEMM Study in the Private Healthcare Sector
Source: Curr Oncol. 2025 Dec 29;33(1):16. doi: 10.3390/curroncol33010016 (PMC12840273; doi:10.3390/curroncol33010016)
Supplement: Supplementary file 1 [file curroncol-33-00016-s001.zip › curroncol-3968190-supplementary.pdf]

Emerging Real-world Treatment Patterns and Clinical Outcomes of Multiple Myeloma in Argentina and Brazil: Insights From the TOTEMM Study in the Private Healthcare Sector

Target journal: *Current Oncology*
Lead Author: Hungria

Supplement

Table S1. Health terms related to MM

Terms in English	Terms in Spanish
Bone marrow biopsy	Biopsia medula osea
Hypogammaglobulinemia	Hipogammaglobulinemia
Myeloma	Mieloma
MM	MM
Autologous bone marrow transplant	Tx autologo medula osea, Trasplante autologo de medula osea, Trasplante autólogo de médula ósea, Tx aut medula osea, Tx autologo de m.o
Multiple myeloma	Mieloma multiple
Autologous HPC transplant	Tx autólogo de CPH, trasplante autólogo de CPH
Multiple myeloma relapse	Recaida de mieloma multiple
Autologous bone marrow transplant status	Estado de trasplante autologo de medula osea
Autologous transplant	Trasplante autologo
Advanced multiple myeloma	Mieloma multiple avanzado
Suspected multiple myeloma	Sospecha de mieloma multiple
Allogeneic bone marrow transplant	Trasplante alogénico relacionado de médula ósea, Trasplante alogenico de medula osea con donante relacionado
Hypogammaglobulinemia with monoclonal band	Hipogammaglobulinemia con banda monoclonal
Hypogammaglobulin	Hipogamaglobulina
Multiple myeloma by IgG	Mieloma multiple por inmunoglobulina G de cadena liviana kap
Bone marrow transplant status	Estado de trasplante de medula osea
IgG myeloma	Mieloma por IgG

IgG = immunoglobulin G; HPC = hematopoietic progenitor cells; MM = multiple myeloma; Tx = transplant.

Table S2. Treatment agents indicated for MM treatment.

Type	Class	Drug
Core MM drugs	Anti-CD38 mAb	Daratumumab
	Anti-CD38 mAb	Isatuximab-irfc
	CS1/SLAMF7 mAb	Elotuzumab
	BCMA-directed mAb	Elranatamab
	BCMA-directed mAb	Teclistamab
	XPO1 inhibitor	Selinexor
	IMiD	Lenalidomide
	IMiD	Pomalidomide
	IMiD	Thalidomide
	PI	Bortezomib
	PI	Carfilzomib
	PI	Ixazomib
Steroids	CS	Dexamethasone
	CS	Methylprednisolone
	CS	Prednisolone
	CS	Prednisone
Other MM drugs	Alkylating agent	Bendamustine
	Alkylating agent	Cyclophosphamide
	Alkylating agent	Melphalan
	Alkylating agent	Vincristine
	Anthracycline topoisomerase inhibitor	Doxorubicin
	Platinum-based chemotherapy	Cisplatin
	Topoisomerase inhibitor	Etoposide

BCMA = B-cell maturation antigen; CTX = chemotherapy; CS = corticosteroid; CS1 = membrane glycoprotein; IMiD = immunomodulatory drugs; mAb = monoclonal antibody; MM = multiple myeloma; PI = proteasome inhibitor; SLAMF7 = signaling lymphocytic activation molecule family member 7; XPO1 = nuclear export protein exportin 1.

Table S3. Variables related to treatment outcomes.

Variable	Definition
Duration of treatment	Period from the start date of the index line through the last treatment episode in the index line
Follow-up period	Time from index date up to death or loss to follow-up or end of study period
Number of lines of therapy	The number of lines of therapy received per patient
Progression-free survival	The estimated progression-free survival time was calculated from beginning of the treatment in the line of therapy to first progression (new antineoplastic drug or death)
Overall survival	Time from index date until death from any cause, including censoring time by loss to follow-up
Relapse	A relapse was considered a progression among incident cases that had ≥2 lines of therapy and ≥1 core drug during follow-up
Rechallenge	Use of the same drug in the subsequent line of therapy
Time to first treatment	Period between the index date and the date of the initial agent in the first line
Time to next treatment	Interval between treatment initiation and initiation of the next treatment (among patients who start the next line of therapy)

Table S4. Algorithm for identifying LOT based on treatment dates and clinical guidelines.

**TOTEMM-A* and TOTEMM-B†**
• The date of first dispensation after index date for any antineoplastic drug was considered the treatment initiation date and marks the beginning of the identification period of the first drug LOT.<br>• All antineoplastic drugs received during the initial 30-day period constituted the drug LOT regimen, although adjustment therapy could have occurred because of toxicity or adverse events after the first cycle.
• The drug regimen included multiple cycles of treatment with the same agent(s) if there was no gap of >90 days between cycles.
• The last day of the drug LOT was the date of the last administration claim (for injectable medications) or the date of the last prescription claim plus 30 days' supply (for oral antineoplastic drugs extended to corticoids).<br>• If the patient did not have 2L data, the end of 1L was defined as the end of follow-up, end of data availability (censoring), or death date, whichever occurred first.<br>• The extension of 30 days of the LOT was applied with caution because this extension could not overlap with the start of the next LOT (described below). In case of overlap, the extension due to oral administration could be <30 days and the end of LOT with oral administration finished 1 day prior starting the subsequent line.
• The start of 2L was defined as the earliest of the following:<br>o Initiation of a new antineoplastic agent that was not part of the prior LOT.<br>o Discontinuation: presence of a ≥90-day gap in all components of LOT after the end of the grace period. The discontinuation of one agent in a LOT did not terminate a LOT if other agents from the LOT were continually prescribed.<br>o Switch: presence of a claim for a new MM therapy following the 1L initiation period, which was not a previously included component of 1L.
• For 2L, 3L, and 4L+, in the absence of either a switch or discontinuation, LOT were considered terminated at the earliest of either death or loss to follow-up or if the LOT continued until the end of study period (censoring).
• Maintenance regimens were subjected to the existing LOT rules; they were considered part of the treatment line they follow.
• Substitution of the reference product for a biosimilar or vice versa did not advance the LOT (e.g., trastuzumab and trastuzumab-anns; rituximab and rituximab/hyaluronidase copack; daratumumab and daratumumab/hyaluronidase). The line name included only the drug with longer duration.
• Megestrol, mesna, plerixafor, radiotherapy, and surgery were excluded from the LOT construction.<br>• Oral, intravenous, or intramuscular steroids were considered part of the LOT, but they did not advance or start the drug LOT.<br>• Ointment and inhaled and ophthalmologic steroids were not considered.

*Hospital Italiano de Buenos Aires, Argentina; January 1, 2018–May 31, 2024. †Orizon, Brazil; January 1, 2018–February 28, 2024.

1L/2L/3L/4L = first/second/third/fourth line; LOT = line(s) of therapy; MM = multiple myeloma; TOTEMM = **T**reatment practices and clinical **o**utcomes in pa**ti**ents with **MM**.

Table S5. 1L–4L treatments in transplant-ineligible patients with MM among incident cases in TOTEMM-A*

	1L		2L		3L		4L	
**Combination/Class**	**N = 72**	**%**	**N = 34**	**%**	**N = 23**	**%**	**N = 10**	**%**
**Monotherapy**	**—**	**—**	**7**	**20.6**	**6**	**26.1**	**1**	**10.0**
IMiD	—	—	3	42.9	3	50.0	—	—
CTX	—	—	3	42.9	3	50.0	1	100.0
PI	—	—	1	14.3	—	—	—	—
**Doublet therapy**	**21**	**29.2**	**19**	**55.9**	**8**	**34.8**	**6**	**60.0**
PI + CS†	15	71.4	3	15.8	2	25.0	1	16.7
IMiD + CS†	3	14.3	6	31.6	3	37.5	1	16.7
CTX + CS†	3	14.3	7	36.8	3	37.5	3	50.0
Anti-CD38 mAb + CS†	—	—	3	15.8	—	—	—	—
BCMA-directed mAb + CS†	—	—	—	—	—	—	1	16.7
**Triplet therapy**	**44**	**61.1**	**8**	**23.5**	**5**	**21.7**	**2**	**20.0**
PI + CTX + CS†	22	50.0	4	50.0	2	40.0	—	—
PI + IMiD + CS†	16	36.4	3	37.5	1	20.0	1	50.0
IMiD + CTX + CS†	3	6.8	—	—	2	40.0	—	—
Anti-CD38 mAb + IMiD + CS†	3	6.8	1	12.5	—	—	—	—
Anti-CD38 mAb + CTX + CS†	—	—	—	—	—	—	1	50.0
**Quadruplet therapy**	**7**	**9.7**	**—**	**—**	**4**	**17.4**	**1**	**10.0**
Anti-CD38 mAb + PI + CTX + CS†	3	42.9	—	—	1	25.0	—	—
PI + IMiD + CTX + CS†	2	28.6	—	—	—	—	—	—
Anti-CD38 mAb + PI + IMiD + CS†	2	28.6	—	—	2	50.0	1	100.0
Anti-CD38 mAb + IMiD + CTX + CS†	—	—	—	—	1	25.0	—	—

*Hospital Italiano de Buenos Aires, Argentina; January 1, 2018–May 31, 2024. †The drugs considered CS were dexamethasone, methylprednisolone, prednisone, and prednisolone. BCMA = B-cell maturation antigen; CS = corticosteroid; CTX = chemotherapy; IMiD = immunomodulatory drug; 1L/2L/3L/4L = first/second/third/fourth line; mAb = monoclonal antibody; MM = multiple myeloma; PI = proteasome inhibitor; TOTEMM = **T**reatment practices and clinical **o**utcomes in pa**ti**ents with **MM**.

Table S6. 1L–4L treatments in transplant-ineligible patients with MM among incident cases in TOTEMM-B*

	1L		2L		3L		4L	
**Combination/Class**	**N = 892**	**%**	**N = 518**	**%**	**N = 263**	**%**	**N = 107**	**%**
**Monotherapy**	**102**	**11.4**	**159**	**30.7**	**78**	**29.7**	**34**	**31.8**
PI	62	60.8	25	15.7	13	16.7	6	17.6
IMiD	32	31.4	84	52.8	53	67.9	23	67.6
CTX	4	3.9	42	26.4	11	14.1	4	11.8
Anti-CD38 mAb	3	2.9	6	3.8	1	1.3	1	2.9
CS1/SLAM7 mAb	1	1.0	1	0.6	—	—	—	—
**Doublet therapy**	**218**	**24.4**	**153**	**29.5**	**91**	**34.6**	**35**	**32.7**
PI + CS†	88	40.4	29	19.0	23	25.3	9	25.7
Anti-CD38 mAb + CS†	40	18.3	29	19.0	12	13.2	6	17.1
PI + IMiD	26	11.9	12	7.8	11	12.1	3	8.6
PI + CTX	24	11.0	7	4.6	4	4.4	6	17.1
Anti-CD38 mAb + PI	19	8.7	21	13.7	8	8.8	—	—
IMiD + CS†	10	4.6	13	8.5	17	18.7	2	5.7
CTX + CS†	6	2.8	34	22.2	8	8.8	3	8.6
Anti-CD38 mAb + IMiD	4	1.8	3	2.0	4	4.4	2	5.7
Other combinations	1	0.5	4	2.6	4	4.4	4	11.4
Anti-CD38 mAb + CTX	—	—	1	0.7	—	—	—	—
**Triplet therapy**	**503**	**56.4**	**180**	**34.7**	**90**	**34.2**	**32**	**29.9**
Anti-CD38 mAb + PI + CS†	209	41.6	95	52.8	30	33.3	12	37.5
PI + CTX + CS†	189	37.6	23	12.8	16	17.8	3	9.4
PI + IMiD + CS†	64	12.7	27	15.0	23	25.6	8	25.0
Anti-CD38 mAb + IMiD + CS†	32	6.4	31	17.2	19	21.1	9	28.1
Anti-CD38 mAb + IMiD + PI	4	0.8	—	—	1	1.1	—	—
Anti-CD38 mAb + CTX + CS†	2	0.4	1	0.6	—	—	—	—
PI + IMiD + CTX	1	0.2	—	—	—	—	—	—
Anti-CD38 mAb + PI + CTX	1	0.2	1	0.6	—	—	—	—
IMiD + CTX + CS†	1	0.2	—	—	—	—	—	—
Other combinations	—	—	2	1.2	1	1.1	—	—
**Quadruplet therapy**	**68**	**7.6**	**25**	**4.8**	**4**	**1.5**	**6**	**5.6**
Anti-CD38 mAb + PI + CTX + CS†	47	69.1	17	68.0	1	25.0	3	50.0
Anti-CD38 mAb + PI + IMiD + CS†	18	26.5	7	28.0	3	75.0	2	33.3
PI + IMiD + CTX + CS†	3	4.4	1	0.2	—	—	—	—

Other combinations	—	—	—	—	—	—	1	16.7

*Hospital Italiano de Buenos Aires, Argentina; January 1, 2018–May 31, 2024. †The drugs considered CS were dexamethasone, methylprednisolone, prednisone, and prednisolone. BCMA = B-cell maturation antigen; CS = corticosteroid; CTX = chemotherapy; IMiD = immunomodulatory drug; 1L/2L/3L/4L = first/second/third/fourth line; mAb = monoclonal antibody; MM = multiple myeloma; PI = proteasome inhibitor; TOTEMM = **T**reatment practices and clinical **o**utcomes in pa**tie**nts with **MM**.

Table S7. Core 1L–4L MM drug use in transplant-ineligible patients with MM among incident cases in TOTEMM-A* and TOTEMM-B*.

		1L		2L		3L		4L	
**Class**	**Drug use alone or in combination with other drugs**	**TOTEMM-A N = 72**	**TOTEMM-B N = 892**	**TOTEMM-A N = 34**	**TOTEMM-B N = 518**	**TOTEMM-A N = 23**	**TOTEMM-B N = 263**	**TOTEMM-A N = 10**	**TOTEMM-B N = 107**
PI	Bortezomib, n (%)	60 (83.3)	718 (80.5)	9 (26.5)	199 (38.4)	7 (30.4)	66 (25.1)	3 (30.0)	23 (21.5)
	Carfilzomib, n (%)	—	38 (4.3)	2 (5.9)	59 (11.4)	1 (4.3)	61 (23.2)	—	26 (24.3)
	Ixazomib, n (%)	—	6 (0.7)	2 (5.9)	9 (1.7)	1 (4.3)	8 (3.0)	—	5 (4.7)
IMiD	Lenalidomide, n (%)	24 (33.3)	196 (22.0)	13 (38.2)	182 (35.1)	10 (43.5)	129 (49.0)	2 (20.0)	49 (45.8)
	Pomalidomide, n (%)	1 (1.4)	—	—	—	2 (8.7)	2 (0.8)	1 (10.0)	—
	Thalidomide, n (%)	4 (5.6)	—	—	—	—	—	—	—
Anti-CD38 mAb	Daratumumab, n (%)	8 (11.1)	378 (42.4)	4 (11.8)	211 (40.7)	4 (17.4)	77 (29.3)	2 (20.0)	32 (29.9)
	Isatuximab, n (%)	—	2 (0.2)	—	2 (0.4)	—	2 (0.8)	1 (10.0)	3 (2.8)
BCMA-directed mAb	Elranatamab, n (%)	—	—	—	—	—	—	1 (10.0)	—
	Teclistamab, n (%)	—	—	—	3 (0.6)	—	2 (0.8)	—	1 (0.9)
CS1/SLAMF7 mAb	Elotuzumab, n (%)	—	2 (0.2)	—	5 (1.0)	—	3 (1.1)	—	4 (3.7)

Values do not sum to 100%, as the data reflect only the number of patients who received a drug relative to the total number of patients within the respective LOT.
*Hospital Italiano de Buenos Aires, Argentina; January 1, 2018–May 31, 2024. †Orizon, Brazil; January 1, 2018–February 28, 2024. BCMA = B-cell maturation antigen; CS1 = membrane glycoprotein; IMiD = immunomodulatory drugs; 1L/2L/3L/4L = first/second/third/fourth line; LOT = line of therapy; mAb = monoclonal antibody; MM = multiple myeloma; PI = proteasome inhibitor; SLAMF7 = signaling lymphocytic activation molecule family member 7; TOTEMM = **T**reatment practices and clinical **o**utcomes in pa**t**i**e**nts with **MM**.

Table S8. Mortality and OS of transplant-ineligible patients with MM among incident cases.

	TOTEMM-A* N = 72	TOTEMM-B† N = 892
**Patients who died, n (%)**	**30 (41.7%)**	**156 (17.5%)**
**Time from index to death, months**		
Median (IQR)	16.8 (31.9)	11.9 (22.3)
**Overall survival**		
Time at risk	1932.3	23,038.5
Incidence rate, per 1000	15.5	6.8
**Survival time, months**		
P25	18.2	44.7
P50	48.8	—
P75	—	—

*Hospital Italiano de Buenos Aires, Argentina; January 1, 2018–May 31, 2024. †Orizon, Brazil; January 1, 2018–February 28, 2024. IQR = interquartile range; MM = multiple myeloma; OS = overall survival; SD: standard deviation; P25/50/75 = percentile 25/50/75; TOTEMM = **T**reatment practices and clinical **o**utcomes in pa**tie**nts with **MM**.

Table S9. OS of transplant-ineligible patients with MM for all incident cases per year of follow-up.

	TOTEMM-A*				TOTEMM-B†			
**Year**	**n**	**Patients who died, n**	**Patients lost to follow-up, n**	**Cumulative survival rate, % (95% CI)**	**n**	**Patients who died, n**	**Patients lost to follow-up, n**	**Cumulative survival rate, % (95% CI)**
**0–1**	72	13	11	80.5 (68.7–88.2)	892	78	191	90.2 (87.9–92.1)
**1–2**	48	7	12	67.0 (53.4–77.5)	623	32	199	84.7 (81.8–87.2)
**2–3**	29	3	2	59.9 (45.4–71.6)	392	26	115	78.1 (74.4–81.4)
**3–4**	24	4	3	49.2 (34.3–62.6)	251	12	94	73.5 (69.1–77.4)
**4–5**	17	3	7	38.3 (23.0–53.4)	145	4	75	70.8 (65.7–75.2)

*Hospital Italiano de Buenos Aires, Argentina; January 1, 2018–May 31, 2024. †Orizon, Brazil; January 1, 2018–February 28, 2024. CI = confidence interval; MM = multiple myeloma; OS = overall survival; TOTEMM = **T**reatment practices and clinical **o**utcomes in pa**tie**nts with **MM**.

Figure S1. Treatment combinations across LOT for transplant-ineligible patients with MM.

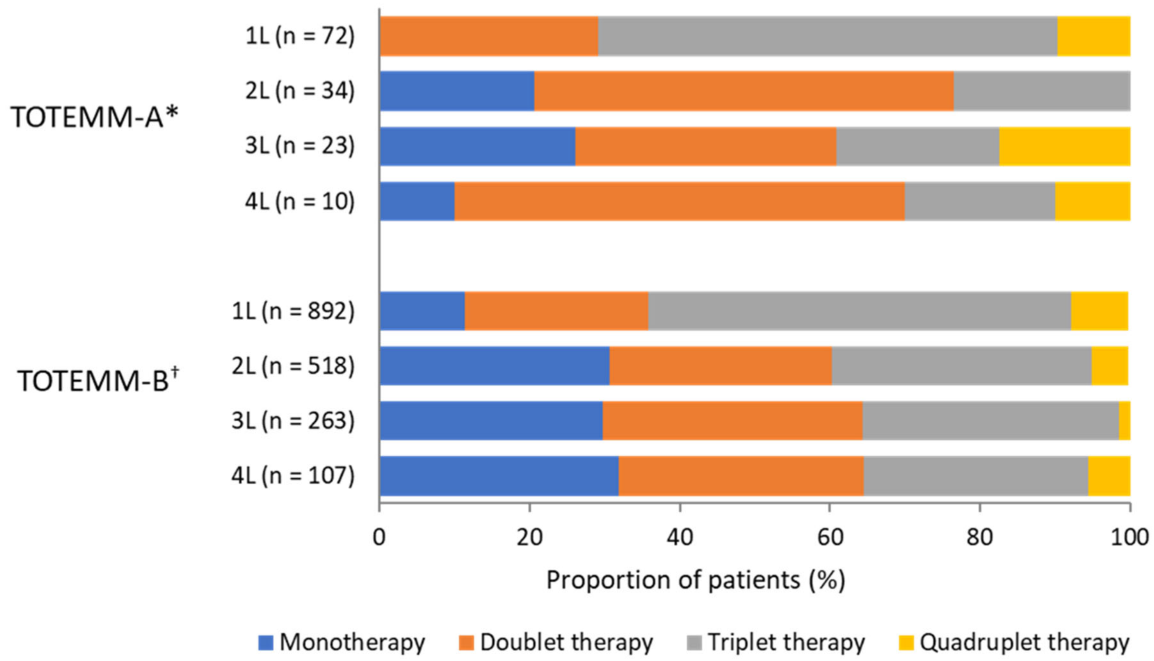

*Hospital Italiano de Buenos Aires, Argentina; January 1, 2018–May 31, 2024. †Orizon, Brazil; January 1, 2018–February 28, 2024. 1L/2L/3L/4L = first/second/third/fourth line; LOT = lines of therapy; MM = multiple myeloma; TOTEMM = **T**reatment practices and clinical **o**utcomes in pa**tie**nts with **MM**.
